# Analysis of the IgE- and IgG-reactivity profiles of asthmatic and non-asthmatic HDM-allergic patients using the ISAC microarray system

**DOI:** 10.1186/2045-7022-4-S2-O17

**Published:** 2014-03-17

**Authors:** Yvonne Resch, Sven Michel, Michael Kabesch, Rudolf Valenta, Susanne Vrtala

**Affiliations:** 1 Medical University of Vienna, Department of Pathophysiology and Allergy Research, Vienna, Austria; 2 Hannover Medical School, Dep. of Ped. Pneumology, Allergy and Neonatology, Hannover, Germany; 3 University Children`s Hospital Regensburg (KUNO) Department of Pediatric Pneumology and Allergy, Regensburg, Germany; 4 Medical University of Vienna, CD Lab. for the Development of Allergen Chips, Vienna, Austria

## Background

House dust mites (HDM) represent one of the most important inducers for respiratory allergies worldwide. The aim of this study was to investigate the IgE- and IgG-reactivity profiles of HDM-allergic individuals suffering only from allergic rhinitis or from allergic asthma.

## Methods

This study included sera from clinically well characterized asthmatic (n=105) and non-asthmatic (n=53) HDM-allergic patients. IgE- and IgG-reactivity to seven HDM-allergens (nDer p 1, rDer p 2, rDer p 5, rDer p 7, rDer p 10, rDer p 21 and rDer p 23) were measured using a customized allergen microarray (i.e., ISAC chip, Thermofisher, Vienna, Austria).

## Results

HDM-allergic individuals suffering from asthma showed striking differences regarding their IgE reactivity profiles compared to the non-asthmatic group. First, the frequency of IgE reactivity to the tested HDM allergens was up to 3-fold higher in the asthmatic than in the non-asthmatic group. Seventy percent of the asthmatics reacted with 3 to 6 of the tested allergens compared to only 45% in the non-asthmatic group. Furthermore, IgE-levels to nDer p 1, rDer p 2, rDer p 5 and rDer p 23 were significantly higher in the asthmatic group. In contrast, HDM-allergic asthma patients showed a lower IgG-binding frequency to the seven HDM allergens than HDM-allergic patients without asthma.

## Conclusion

The IgE and IgG reactivity profiles to HDM allergens differ considerably in patients with mild (i.e., rhinitis) and severe (i.e., asthma) respiratory symptoms due to HDM allergy.

